# Constraint-induced movement therapy following stroke: a commentary

**DOI:** 10.12968/bjnn.2024.0041

**Published:** 2024-12-02

**Authors:** D Lamb, JE Hill, R Santos

**Affiliations:** North Cumbria Integrated Care NHS Foundation Trust; University of Lancashire

## Abstract

Over 113,000 patients present with stroke each year in the United Kingdom. The societal cost of which is approximately £26 billion annually, with £20.6 billion attributed to ongoing care. Approximately 70% of stroke survivors suffer from impaired arm function, with recovery patterns heavily influenced by initial motor weakness. Constraint-induced movement therapy (CIMT) has demonstrated some potential in rehabilitating this dysfunction where finger extension is preserved. CIMT is therefore now recommended in national healthcare guidelines. Systematic reviews of CIMT have varied in their sample groups, focusing on acute, subacute and chronic strokes, with varying CIMT delivery protocols. A recent systematic review was undertaken by Yang et al (2023) with the aim of identifying the efficiency of CIMT in patients with preservation of finger extension and the optimum protocol for delivery. This commentary aims to critically appraise the methods used within the review by Yang et al., (2023) and expand upon the findings in the context of clinical practice.

## Introduction

Stroke is the third most common cause of disability worldwide (Campbell and Khatri 2020). In the United Kingdom (UK) over 113,000 patients present with stroke each year, eighty-eight percent of which experience contralesional motor weakness (Rothwell et al. 2004). The annual cost of stroke in the UK is estimated at £26 billion, with £20.6 billion relating to ongoing care (Patel et al., 2020). The future incidence of stroke is projected to rise, making effective rehabilitation a clinical priority (Stroke Association 2016).

Approximately 70% of stroke patients experience loss of arm function (Lieshout et al. 2020). Patterns of arm recovery are varied and largely dependent on the initial degree of weakness and patency of the corticospinal tract (Coupar et al., 2012). An early indicator of positive upper limb functional recovery is the preservation or return of finger/wrist extension (Stinear et al. 2017). Maximising recovery requires an effective rehabilitation approach, methods of which are varied globally (Pollock et al. 2014).

Constraint-Induced Movement Therapy (CIMT) is a rehabilitative strategy aimed at improving the functional use of an effected limb after stroke (Kwakkel et al. 2015). The original CIMT evidence involved a two to three-week programme where the unaffected limb is restrained for 90% of waking hours (Reiss et al. 2012). While the unaffected limb is restrained, six hours of graded exercises and functional tasks are practiced per day with the affected limb (MorrisTaub and Mark 2006).

CIMT is recommended in the UK (National clinical guidelines for stroke 2023) stating “People with stroke who have at least 20 degrees active wrist extension and 10 degrees of active finger extension should be considered for constraint induced movement therapy”. In alignment with these guidelines, Yang et al., (2023) recently conducted a systematic review to evaluate the impact of CIMT in patients with intact cognitive function and preserved finger extension. The review also aimed to determine the most effective protocol for administering CIMT.

### Aim of commentary

This commentary aims to critically appraise the methods used within the review by Yang et al., (2023) and expand upon the findings in the context of clinical practice.

### Critical Appraisal and Methods of Yang et al., (2023)

Using the Measurement Tool to Assess Systematic Reviews (AMSTAR 2), this systematic review was judged to achieve 11 out of 16 criteria (Shea et al. 2017). See Table 1 for critical appraisal and key methodological processes.

The main areas of methodological concern pertained to the search terms used within the primary search strategy. A relatively narrow set of terms were used regarding the condition. While both ischemic and haemorrhagic stroke types are included in the inclusion criteria, the latter term was not part of the search strategy. Additionally, terms such as “cerebral vascular accident” and “intracerebral haemorrhage” were also not included in the search strategy. It is important when undertaking a search to include all relevant terms which may be used within the literature (Bramer et al. 2018). When key search terms are missed, important studies might be omitted from the systematic review, potentially affecting the overall effect estimates.

The studies included and excluded from the review are identified in the supporting information, however no further justification for either inclusion or exclusion is given. For transparency and repeatability, it is important that systematic reviews present all excluded studies along with the justification for their exclusion (Schmidt et al. 2014). The justification for including only randomized controlled trials was insufficient. It is typically recommended to include the highest quality of evidence in effectiveness systematic reviews, which ideally are RCTs in most cases (Higgins et al. 2023). Therefore, it is quite common for systematic reviews not to justify the specific inclusion criteria of only including RCTs.

The funding sources of the studies included within the systematic review were not reported upon by the authors. Historically the influence of funding has been identified as a possible methodological issue regarding selective reporting, results suppression and fraud (Resnik 2000). This lack of reporting of funding may be further impacted due to the lack of assessment of publication bias. There was a limited number of studies which makes statistical methods of assessing publication bias underpowered (van AertWicherts and van Assen 2019). However alternative methods such as comparison of protocol registries compared to current publications could have been undertaken (NORRIS et al. 2012). This review did not assess the impact of various issues of risk of bias of the included studies on the estimates for the outcomes presented. This type of subgroup analysis allows assessment of particular issues such as whether the high risk of bias identified in the included studies may have an effect on the estimates presented (Higgins et al. 2023). In summary this systematic review provides a comprehensive summary of outcomes, however these methodological issues should be taken into consideration when interpreting the certainty in the estimates presented in the context of specific practice scenarios.

### Main findings of Yang et al., (2023)

After duplicate removal only 428 citations were identified, six of which were included in the systematic review (n = 169). Of the six, only one was a low risk of bias, while the remaining five studies exhibited some criteria resulting in unclear or high risk of bias.

Using a fixed effects model there was a statistical increase in motor function test (WMFT_FA) scores when comparing CIMT to usual care (n = 94, Means difference: 0.5, 95% confidence interval [Cl] 0.21 to 0.80, I^2^ = 0%). Using a random effects model there was no evidence of difference for Motor Activity Log - Amount of Use (MAL_AOU) [I^2^ = 81%] and Motor Activity Log - Quality of Movement (MAL_QOL) [I^2^ = 76%]), Wolf Motor Functional Test-Performance Time (WMFT_PT) [I^2^ = 81%] and Fugl-Meyer assessment scores [I^2^ = 51%]. For these four outcomes there was moderate to substantial heterogeneity.

Subgroup analysis included four studies with chronic stroke symptoms lasting over six months. When synthesized through a random effects model, there was a statistically significant increase in the MAL_AOU when comparing CIMT to usual care (Standard means difference [SMD]: 0.96, 95% Cl 0.20 – 1.72, I^2^ = 64%). Similarly, a statistically significant large effect was also observed in MAL_QOL (SMD: 1.01, 95% Cl 0.50 – 1.51, I^2^ = 24%) when comparing chronic stroke patients with usual care. There was no evidence of difference for the same subgroup analysis and meta-regression for the outcomes of WMFT_PT, WMFT_FA and Fugle-Meyer.

To evaluate dose response, a subgroup analysis of studies implementing interventions lasting more or less than three hours was undertaken. There was a statistically significant increase observed in WMFT_FA (MD: 0.59, 95% Cl 0.23 to 0.94, I^2^ = 0%) in the two trails delivering more than three hours of CIMT. The continuous measurement of constraint time however was not identified as a statistically significant associated moderating factor in the other four outcomes. Furthermore, there was no evidence of association found for the potential moderating factors of total intervention time and hours per week for all outcomes.

### Commentary

The clinically significant mean change score in WMFT_FA score has been proposed as a 0.2 to 0.4 points for cognitively intact stroke patients (Lin et al. 2009). Thus, Yang et al found a clinically significant change in WMFT_FA of MD 0.5 (95% confidence interval [Cl] 0.21 to 0.80, I^2^ = 0%) in patients receiving CIMT. CIMT you are did not produce a statistically significant improvement in WMFT_PT compared to usual care. However, this measure has previously been identified as less responsive to detecting change in comparison to the WMFT_FA (Lin et al. 2009). The other outcomes appeared to show no evidence of effect, however this may be misleading, as it is important to note that no evidence of effect does not mean evidence of no effect. The confidence intervals for these outcomes remain very wide, indicating that, depending on the effect levels, there could still be clinically significant improvements.

The substantial heterogeneity for the other outcomes is suggestive of important underlying moderating factors that influence the effect of CIMT, investigated within this review by meta-regression. The large, statistically significant effect on the MAL quality and amount of movement scores for patients with stroke symptoms greater than six months is of clinical importance. This finding justifies further exploration of this specific clinical scenario, despite the notable heterogeneity and wide confidence intervals. The timing of implementing CIMT programmes should be a consideration when developing stroke rehabilitation services within a health system such as the National Health Service (NHS). Early mobilisation and Hyper Acute Stroke Rehabilitation has developed significantly within the NHS over the last decade (NHS 2021), whereas evidence of effective therapies in the chronic stage may be a future focus. Further evidence of this moderating factor may allow for greater implementation of CIMT at the most effective point in the stroke journey.

The implementation of CIMT within health services has posed multiple practical and workforce challenges (Daniel et al. 2012). Thus, establishing the minimal dose-response protocol is essential for efficient, cost effective, implementable services (Daniel et al. 2012). Yang et al., (2023) found a clinically significant increase in WMFT functional ability within trials delivering more than 3 hours of therapy per day. No evidence of association however was found for intervention total time and hours per week for all outcomes. A prior systematic review investigating the timing and dosage of rehabilitation for upper limb interventions post-stroke found that the intervention doses and sample sizes of the studies were generally too small to detect clinically significant changes, despite the increasing volume of research in this area (Hayward et al. 2021). In conclusion, this review enhances the evidence that CIMT is effective in improving functional ability following stroke.

The existing literature indicates substantial unexplained heterogeneity for the effects of CIMT on MAL_AOU, MAL_QOL, WMFT_PT and Fugl-Meyer assessment scores, suggesting the presence of unrecognized moderating factors affecting the efficacy of this intervention. To advance the understanding and optimization of CIMT, future research should aim to identify and analyse potential moderating factors that may influence the effectiveness of CIMT, such as patient demographics, stroke severity, time since stroke, and individual variability in response to therapy. Future RCTs should compare multiple possible moderating factors such as dose scenarios of CIMT, varying both the amount and duration of therapy to determine the optimal dosing strategy. Further exploration and effectiveness assessment should be undertaken to explore both possible moderating factors and mediating factors of CIMT for chronic stroke patients. Finally due to the notable methodological issues regarding the search strategy of this review it is recommended that an update of this review is required with a more broader and comprehensive search strategy is to ensure that all relevant studies are identified and included in the review.

### CPD reflective questions

1. At what stage in the rehabilitation journey post stroke would you consider CIMT to be most effective?
2. Should cognitive ability be part of routine screening prior to CIMT, given that establishing MMSE score give greater confidence to the clinician and patient in its possible beneficial effects?
3. The implementation of a daily 6-hour exercise and functional task programme can be challenging in modern day health services, how can this be implemented while maintaining fidelity to the evidence in the NHS?

## Figures and Tables

**Table 1:** Critical appraisal using the AMSTAR 2 tool for assessing systematic review of Yang et al., (2023) and key methodological processes.

	AMSTAR 2 items	Responses/Methods
	**1. Did the research questions and inclusion criteria for the review include the components of PICO?**	Yes - Only randomised controlled clinical trials comparing constraint-induced movement therapy with conventional rehabilitation methods on adult stroke patients were included in this review. The main outcomes of interest were Motor Activity Log (amount of use and quality of movement), the Wolf Motor Function Test (functional ability and performance time) and Fugl-Meyer assessment.
	**2. Did the report of the review contain an explicit statement that the review methods were established prior to the conduct of the review and did the report justify any significant deviations from the protocol?**	Yes - The protocol was registered on the open science framework platform.
	**3. Did the review authors explain their selection of the study designs for inclusion in the review?**	No – No reasoning for inclusion of only randomised controlled trials was given.
	**4. Did the review authors use a comprehensive literature search strategy?**	No - A multi-database search was carried out in January 2022, PubMed, Embase, and the Cochrane Library by reviewing Abstract and titles. Additional data was identified through conference abstracts and reference lists of included studies. However, the search was not rerun within the 12 months before the publication to screen for potential eligible studies during this period. The search was not restricted neither by year nor language, and a relevant set of text words was used to define the search in each database.
	**5. Did the review authors perform the study selection in duplicate?**	Yes – For the eligibility of the papers, the abstract and title and full-text screening was undertaken by 2 independent reviewers. Disagreements were resolved by discussion.
	**6. Did the review authors perform data extraction in duplicate?**	Yes – Data extraction was undertaken by two reviewers independently.
	**7. Did the review authors provide a list of excluded studies and justify the exclusions?**	Partially – A list of excluded studies was supplied however no justification for exclusion was given.
	**8. Did the review authors describe the included studies in adequate details?**	Yes – A table of characteristics of included studies was presented.
	**9. Did the review authors use a satisfactory technique for assessing the risk of bias in the individual studies that were included in the review?**	Yes – The reviewers assessed the risk of bias using the Cochrane Risk of Bias (RoB1) tool.
	**10. Did the review authors report on the sources of funding for the studies included in the review?**	No – The review did not indicate where the studies received their funding from.
	**11. If meta-analysis was performed did the review authors use appropriate methods for statistical combination of results?**	Yes – The effectiveness of the intervention on the three outcomes was reported using standardized mean difference for the first outcome and mean difference for the last two, with 95% confidence intervals. Heterogeneity was assessed using the I square (I2) statistic and Cochran’s Q test.
		Subgroup analyses, for differences in post-stroke duration (chronic and subacute phase) and length of constraint time (more or less than 3 h) were undertaken using a mixed-effects linear meta-regression model.
	**12. If meta-analysis was performed did the review authors assess the potential impact of RoB in individual studies on the results of the -analysis or other evidence synthesis?**	No – No subgroup analysis was undertaken regarding the possible impact of risk of bias.
	**13. Did the review meta-authors account for RoB in individual studies when interpreting/discussing the results of the review?**	Yes – There was some discussion regarding methodological issues. It is debatable if full interpretation of the overall risk of bias was applied to the interpretation of the findings. No structured method such as GRADE criteria were applied.
	**14. Did the review authors provide a satisfactory explanation for and discussion of any heterogeneity observed in the results of the review?**	Yes – Both subgroup analysis and meta-regression was undertaken to explore the reasons of heterogeneity.
	**15. If they performed quantitative synthesis did the review authors carry out an adequate investigation of publication bias (small study bias) and discuss its likely impact on the results of the review?**	No – Method of assessment of publication bias was undertaken.
	**16. Did the review authors report any potential sources of conflict of interest, including any funding they received for conducting the review?**	Yes – The review declared the funding organisation of Tri-Service General Hospital.

## Key Points

- Evidence suggests that Constraint-Induced Movement Therapy (CIMT) may have a moderate effect on Wolf Motor Function Test Functional Ability scores.
- The effects of CIMT on Motor Activity Log (Amount of Use & Quality of Movement), Wolf Motor Function Test (Performance Time), and Fugl-Meyer Assessment scores remain inconclusive.
- Future research should explore important moderating factors affecting the effectiveness of CIMT on Motor Activity Log (Amount of Use & Quality of Movement), Wolf Motor Function Test (Performance Time), and Fugl-Meyer Assessment scores.
